# The Role of ARX in Human Pancreatic Endocrine Specification

**DOI:** 10.1371/journal.pone.0144100

**Published:** 2015-12-03

**Authors:** Blair K. Gage, Ali Asadi, Robert K. Baker, Travis D. Webber, Rennian Wang, Masayuki Itoh, Masaharu Hayashi, Rie Miyata, Takumi Akashi, Timothy J. Kieffer

**Affiliations:** 1 Department of Cellular and Physiological Sciences, University of British Columbia, Vancouver, British Columbia, Canada; 2 Departments of Physiology, Pharmacology & Medicine, Child Health Research Institute, the University of Western Ontario, London, Ontario, Canada; 3 National Center of Neurology and Psychiatry, Tokyo, Japan; 4 Tokyo Metropolitan Institute of Medical Science, Tokyo, Japan; 5 Tokyo Medical and Dental University, Tokyo, Japan; 6 Department of Surgery, University of British Columbia, Vancouver, British Columbia, Canada; INSERM UMRS 1138, FRANCE

## Abstract

The *in vitro* differentiation of human embryonic stem cells (hESCs) offers a model system to explore human development. Humans with mutations in the transcription factor Aristaless Related Homeobox (ARX) often suffer from the syndrome X-linked lissencephaly with ambiguous genitalia (XLAG), affecting many cell types including those of the pancreas. Indeed, XLAG pancreatic islets lack glucagon and pancreatic polypeptide-positive cells but retain somatostatin, insulin, and ghrelin-positive cells. To further examine the role of ARX in human pancreatic endocrine development, we utilized genomic editing in hESCs to generate deletions in *ARX*. ARX knockout hESCs retained pancreatic differentiation capacity and ARX knockout endocrine cells were biased toward somatostatin-positive cells (94% of endocrine cells) with reduced pancreatic polypeptide (rarely detected), glucagon (90% reduced) and insulin-positive (65% reduced) lineages. ARX knockout somatostatin-positive cells shared expression patterns with human fetal and adult δ-cells. Differentiated ARX knockout cells upregulated PAX4, NKX2.2, ISL1, HHEX, PCSK1, PCSK2 expression while downregulating PAX6 and IRX2. Re-expression of ARX in ARX knockout pancreatic progenitors reduced HHEX and increased PAX6 and insulin expression following differentiation. Taken together these data suggest that ARX plays a key role in pancreatic endocrine fate specification of pancreatic polypeptide, somatostatin, glucagon and insulin positive cells from hESCs.

## Introduction

Currently the most effective therapy for type 1 diabetes is cadaveric human islet transplantation, although the demand for donor tissue outpaces the availability of cells. Based on this need, the use of pluripotent stem cells and their differentiated progeny is being explored as a potential source of insulin secreting β-cells for transplantation [1]. Many *in vitro* differentiation protocols generate polyhormonal endocrine cells that co-express insulin, glucagon and the transcription factor Aristaless Related Homeobox (ARX) [2–7]. When transplanted, these immature polyhormonal cells generate predominantly α-cells that maintain prominent expression of ARX [2, 8]. The role of ARX in the development of pancreatic endocrine cells from human embryonic stem cells (hESCs) is unclear, but numerous studies have assessed its role in mice and rare human samples.

ARX is expressed in a wide variety of tissues including the brain, heart, skeletal muscle, testis, intestine, and pancreas [9–14]. The human *ARX* gene has five exons that together encode a number of protein domains of the transcription factor. These include a series of poly-alanine repeats whose expansion is associated with multiple seizure phenotypes and Partington syndrome in humans and mice, as well as reduced α-cell specification and increased α-cell apoptosis [15, 16]. Humans with X-linked lissencephaly with ambiguous genitalia (XLAG, OMIM # 300215) represent some of the most severe clinical effects of null mutations in *ARX* through functional loss of the DNA binding prd-like homeodomain [15]. Patients with XLAG lack glucagon and pancreatic polypeptide (PP)-positive cells, while insulin-, somatostatin- and ghrelin-positive cell numbers seemingly remain largely unchanged [17]. Similarly, ARX-deficient mice fail to form glucagon-positive cells, but still form insulin- and somatostatin-positive cells [9]. In mice where *ARX* was overexpressed in various pancreatic lineages (PDX1-, PAX6- or insulin-positive), increased numbers of glucagon- and PP-positive cells were observed at the expense of both the insulin- and somatostatin-positive lineages [18]. Furthermore, PAX4 knockout mice lack insulin- and somatostatin-positive cells but retain numerous glucagon-positive cells [19]. This positive regulation of the α-cell lineage by ARX and β/δ-cell lineage of PAX4 reflects a reciprocal transcriptional repression mechanism between ARX and PAX4. Work by Collombat et al. revealed that ARX represses *PAX4* through a transcriptional enhancer upstream of the *PAX4* gene, whereas PAX4 represses *ARX* transcription by binding to a 3' enhancer of the *ARX* gene [20]. This model of specification of the α- versus β/δ- lineages of pancreatic endocrine cells may also be present in human fetal development, as both PAX4 and ARX are expressed within the same time frame (~8–9 weeks) of gestation [21–23]. In hESC differentiation, ARX/insulin/glucagon co-positive cells generate primarily ARX-positive α-cells following transplantation [2, 8], suggesting that ARX is associated with the early formation of pancreatic polyhormonal cells and subsequently, the glucagon lineage.

To further assess the role of ARX in the specification of human pancreatic endocrine cells, we generated hESCs deficient in ARX and examined pancreatic endocrine development. We found that ARX ko hESCs were able to differentiate similarly to wild-type hESCs. However, endocrine cells derived from ARX ko hESCs expressed very little if any glucagon or PP, thus resembling the pancreatic endocrine populations in human XLAG patients. ARX ko endocrine cells also had low expression of insulin leaving a large population of somatostatin-positive cells. Re-expression of ARX increased the numbers of insulin-positive cells derived from ARX ko hESCs suggesting that during hESC differentiation, ARX is required for the formation of glucagon-, PP-, and insulin-positive cells in this model of human embryonic development.

## Materials and Methods

### Ethics Statement

This work was approved by the Canadian Institute for Health Research Stem Cell Oversight Committee (approval number: 229333) and the University of British Columbia Office of Research Services Clinical Ethics Board (UBC CREB number: H08-01618).

### Culture of hESCs

CA1S cells were a kind gift from Dr. James Piret of the University of British Columbia having been derived from CA1 hESCs [24] (Dr. Andras Nagy, Mount Sinai Hospital, Toronto, Ontario, Canada) and previously described [25]. CA1S hESCs were cultured on 1:30 diluted growth factor reduced-Matrigel (BD Biosciences, Mississauga, ON, Canada) in mTeSR1 media (STEMCELL Technologies, Vancouver, BC, Canada) as previously described [3].

### Genomic Editing of CA1S Cells by Zinc Finger Nuclease

**Fig 1:**
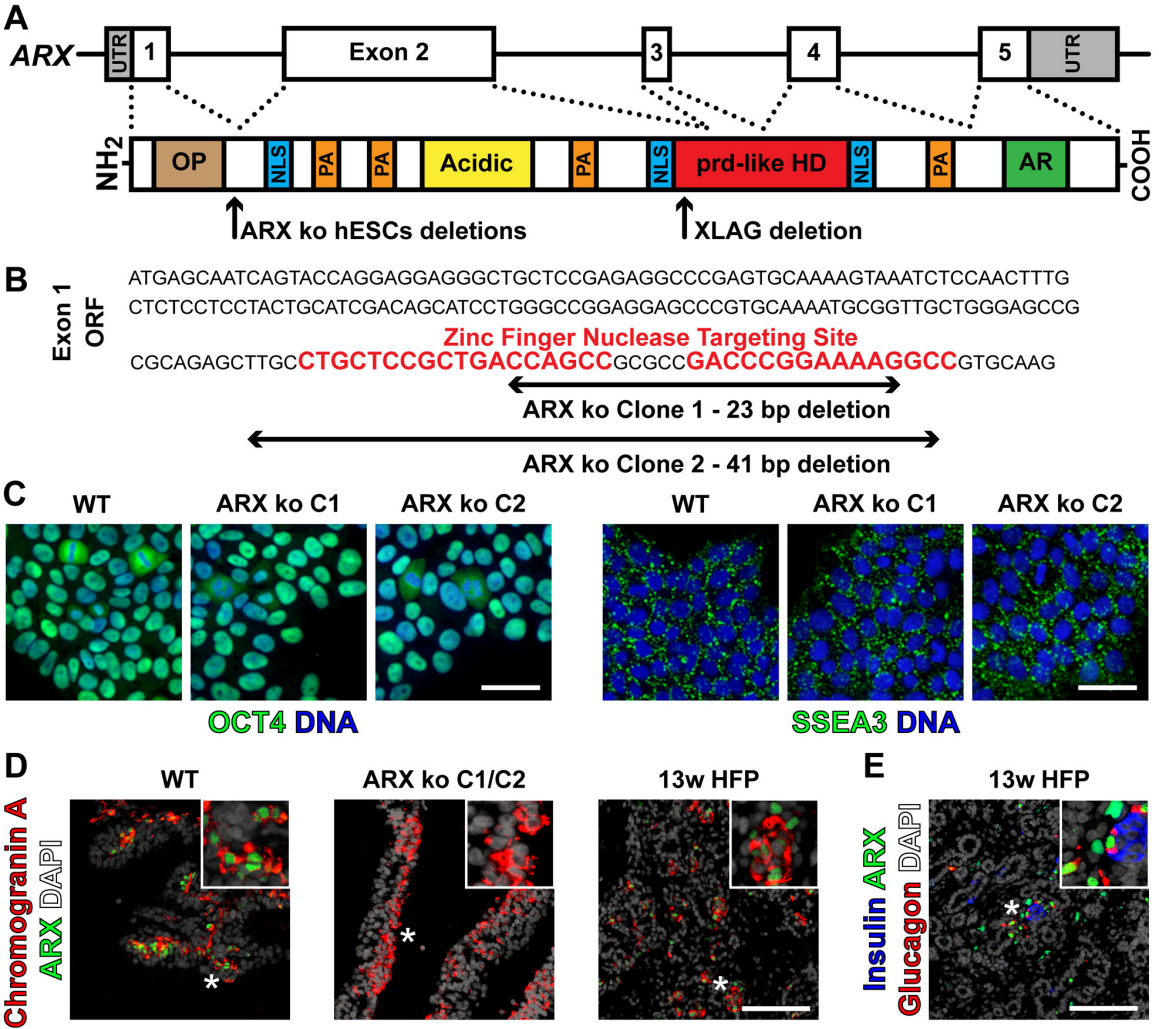
Generation of ARX ko hESCs. (A) Schematic representation of the *ARX* gene and contained exons. Approximate protein domains and important functional regions of ARX including: octapeptide domain (OP), nuclear localization signals (NLS), poly-alanine expansion repeats (PA), Acidic domain (Acidic), prd-like homeodomain (prd-like HD), and Aristaless domain / C-peptide (AR). Approximate location of zinc finger nuclease mediated genomic editing induced deletion mutations in ARX knockout (ARX ko) hESCs and naturally occurring mutation of the XLAG pancreatic sample used in this study are also depicted. (B) Specific nucleotide deletions of *ARX* surrounding the high-specificity 36 bp ZFN recognition sequence within exon 1. ARX ko hESCs clones resulted in a frameshift mutation and premature stop codons in ARX. (C) Wild type (WT) and ARX ko clones 1 and 2 immunostained for pluripotency associated markers OCT4 (green) or SSEA3 (green) and counterstained for DNA with Hoechst 33342. Scale bar is 50 μm. (D) Immunostaining of ARX protein in agarose-embedded sections of 26-day differentiated WT or ARX ko pancreatic cells as well as human fetal pancreatic tissue at 13 weeks of gestation (13w HFP); ARX (green), chromogranin A (red), and DAPI (white). (E) 13w HFP immunostained for ARX (green), insulin (blue), glucagon (red), and DAPI (white) showing natural ARX expression patterns. Inset is a ~3x enlarged portion from the region indicated by the star. Scale bar for D and E is 100 μm.

Subconfluent CA1S hESCs were enzymatically dissociated to 5–25 cell clusters using Accutase (7 min, 37°C, STEMCELL Technologies) and counted using a Scepter^TM^ 2.0 cell counter (Millipore, Billerica, MA, USA). For electroporation of hESCs using a Neon transfection system (Invitrogen, Carlsbad, CA, USA), optimal gene delivery targeting 40–50% of cells was found to be 2 pulses at 1050 V for 30 ms (data not shown) with, 2.5 x 10^5^ cells per 100 μl buffer R volume supplemented with 5 μl Zinc Finger Nuclease encoding mRNA (2 μg per nuclease of the pair) (Catalogue number CKOZFN3556, SIGMA Aldrich, St. Louis, MO, USA). Immediately after electroporation, cells were plated in 2.5 ml mTeSR1 media supplemented with 1 μM Y-27632 (Calbiochem, La Jolla, CA, USA) in a Matrigel coated 6-well plate. After 72 hours of expansion, 576 wells were seeded in 96-well plates as single cells at 0.5 cells /well. Single-colony, hESC-like clones (11.3% of seeded wells) were identified morphologically, enzymatically passaged, and subjected to a PCR-based screen and sequencing of the *ARX* locus nuclease cut site (forward primer 5'-TCAGTACCAGGAGGAGGGC, reverse primer 5'- GAGACAGCCCTGGCTAGATG) to identify deletion clones. Of the identified *ARX* mutated clones (3 of 65), 2 had *ARX* deletions which resulted in small frameshift mutations with premature stop codons without disruption of *in silico* splice donors and were thus chosen for further study (Fig 1; C1 and C2). WT and ARX ko cells were stained for OCT4 and SSEA3 as previously described [3],using antibodies listed in S2 Table.

### Pancreatic Differentiation of hESCs

**Fig 2:**
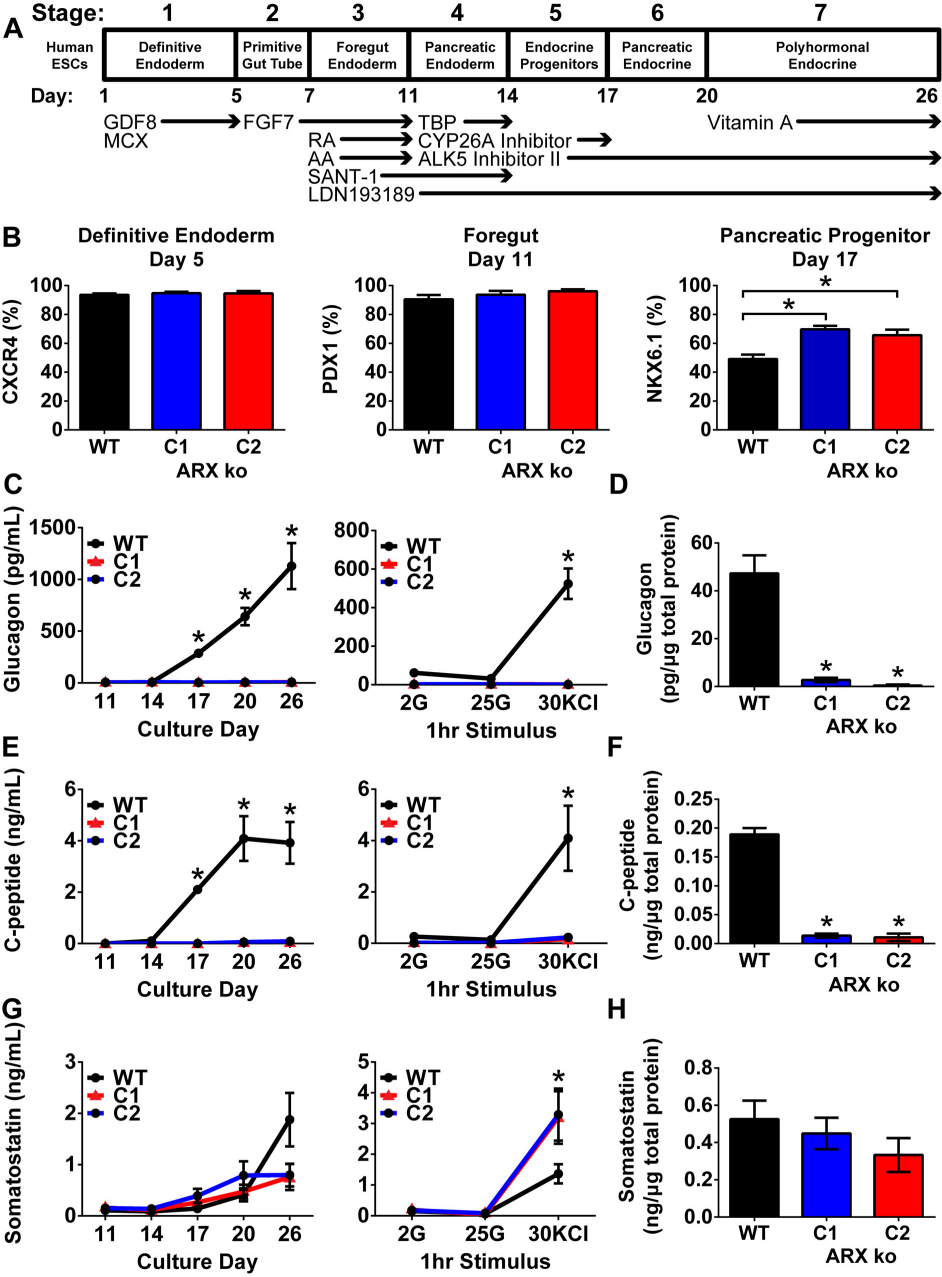
Pancreatic Differentiation of ARX ko hESCs. (A) Schematic representation of the *in vitro* pancreatic endocrine differentiation method as previously described by Bruin et al. (2014). (B) Flow cytometry of wild type (WT), ARX knockout (ARX ko) clone 1 (C1), and ARX ko clone 2 (C2) at the end of definitive endoderm (CXCR4), foregut (PDX1), and pancreatic progenitor (NKX6.1) stages. (C-H) Media composition and cellular content analysis of differentiating wild type and ARX ko hESCs. Glucagon, C-peptide and somatostatin were assayed from un-stimulated media samples collected from days 11 to 26 (C, E, G; left column); a static sequential glucose stimulated hormone release assay was performed on day 24 of culture which included 1 hour treatments of low glucose (2 mM), high glucose (25 mM), and potassium chloride (KCl, 30 mM) (C, E, G; right column). (D, F, H) total hormone content measurements of day 26 WT and ARX ko cell lysate samples normalized for total protein content. (N = 4 independent differentiation trials for all data sets). * indicates p < 0.05 wild type vs ARX ko C1 and C2 at indicated time point or treatment based on a one-way ANOVA and Bonferroni post hoc test.

Ninety percent confluent cultures of CA1S cells were differentiated as previously described in detail [26] and summarized in Fig 2A. At day 5, 11, and 17 of the culture, developing cells were assessed for expression of CXCR4 (definitive endoderm), PDX1 (foregut endoderm), and NKX6.1 (pancreatic endoderm) by flow cytometry (n = 4 independent differentiation trials) as previously described [26] using antibodies described in S2 Table. Hormone content of 24-hour static media samples (end of each stage), static sequential glucose/KCl release (day 24; 1 hour each in 2 mM glucose, 25 mM glucose, 30 mM KCl) and total content (day 26) were assayed by radioimmunoassay as previously described [3] (C-peptide; HCP-20K, glucagon; GL-32K, both Millipore) or enzyme-linked immunoassay (somatostatin; EK-060-03, Phoenix Pharmaceuticals Inc., Burlingame, CA, USA) (n = 3–4 samples for each treatment/timepoint of 4 trials). Total protein samples were prepared by vortexing snap frozen cell pellets in RIPA lysis buffer and total protein content was assessed by BCA assay (Pierce Biotechnology, Rockford, IL, USA).

### Adenoviral *ARX* Cloning and Application

Since the *ARX* gene has high GC content (70–90%) which is empirically resistant to complete reverse transcription, the human *ARX* open reading frame (ORF) was cloned from genomic DNA as individual exons that were seamlessly reassembled using Golden Gate cloning methods [27]. Once fully assembled, the *ARX* ORF was cloned downstream of the human EF1α promoter and its first intron such that the start codon of *ARX* replaced that of EF1α. Subsequently the 3' end of the *ARX* ORF was appended with a SV40 polyadenylation sequence, sequence verified and cloned into the pAdeno-X (Clonetech) vector using *I-CeuI* and *PI-SceI* (New England Biolabs). Complete Ad ARX virions and viral concentrates were generated using HEK293 cells (1 x 10^7^ PFU/ml). Control Ad eGFP virions (1 x 10^9^ PFU/ml) (CMV promoter), were amplified from a kind gift from Dr. P. Robbins.

Adenoviral infection of differentiating hESCs (WT and ARX ko) occurred on days 13 and 19 of differentiation (24 hours, MOI of 2 based on 1 x 10^6^ cells / well). eGFP was visualized using an Axiovert 200 microscope (Carl Zeiss Canada, Toronto, ON, Canada) as previously described [28]. Transgene delivery was assessed 72 hours after infection based on eGFP fluorescence by flow cytometry using a LSRII cytometer (Becton Dickinson, San Jose, CA, USA) and FlowJo Software (Tree Star, Ashland, OR, USA) (n = 3).

### Quantitative Reverse Transcriptase PCR (RT-qPCR)

Snap frozen cell sheets were used in RNA isolation, cDNA synthesis, and RT-qPCR as previously described [3] or in the preparation of cell lysates. RT-qPCR using primers in S1 Table was done in 3–4 biological replicates with gene expression normalized to HPRT then to a reference sample of pooled adult human islet cDNA. Data were quantified by one-way ANOVA with Bonferroni post-hoc tests used to compare like genotypes over time or unlike genotypes at given time points (P<0.05 was considered significant).

### Immunocytochemistry

Various pancreatic tissue samples or differentiated hESCs were immunostained as 5 μm paraffin sections as previously described [3, 29]. Dilutions for primary antibodies can be found in S2 Table. Secondary antibodies were diluted 1:1000 and included Alexafluor-488, -555 and -647. Images for all in-well and slide-based immunofluorescence were captured using an ImageXpress Micro^TM^ automated microscope and associated MetaXpress software (Molecular Devices). Single cell quantification of immunoreactive positive cells was performed using MetaXpress software and associated Multi-Wavelength-Cell-Scoring module, which allows unbiased nuclear and cytoplasmic scoring of cells using user-defined intensity thresholds in a nucleocentric manner. All quantification was performed on 3–4 biological replicates. Data are reported as mean ± standard error of the mean with significance reported as P< 0.05 based on one-way ANOVA with Bonferroni post-hoc analysis.

### Tissue Samples

All human samples were acquired under informed written consent for use in these and other studies and were received with out patient identifying information. Human fetal pancreatic tissue was collected and provided by Dr. R. Wang according to protocols approved by the Health Sciences Research Ethics Board at the University of Western Ontario. XLAG (980–983 del AACA) and age matched control pancreatic tissue were collected and provided by Drs. M. Itoh, M. Hayashi, R. Miyata, and T. Akashi having been previously described [17, 30]. Adult human pancreatic tissue and isolated islets were provided by Drs. G. Warnock and Z. Ao of the Irving K. Barber Human Islet Isolation Laboratory in Vancouver BC. *Arx* ko and WT E18.5 pancreata were provided by Dr. P. Collombat (University of Nice Sophia Antipolis, UFR Sciences, Nice, France).

## Results

### Generation and Validation of ARX ko hESCs

To examine the role of ARX in pancreatic endocrine differentiation, we generated two independent ARX ko hESC clones with genomic deletions in exon 1, such that all protein components from exons 2–5 are lost (Fig 1A). This was accomplished by targeted genomic editing using a zinc-finger nuclease pair that stimulated double stranded DNA breaks in the single copy of *ARX* in male (XY) CA1S hESCs. *ARX* deletion mutants were identified by PCR screening and sequencing including clone 1 (C1) and clone 2 (C2) that contained 23 and 41 base pair frameshift deletions respectively (Fig 1B). Sequencing of the mutations revealed frameshift deletions that resulted in a premature stop codon and a predicted ARX protein that lacked all domains from exons 2–5. Both wild type (WT) and ARX knockout (ARX ko) hESC clones maintained expression of the pluripotency associated factors OCT4 and SSEA3 suggesting that the genomic editing and cloning process did not compromise pluripotency (Fig 1C). To validate the loss of ARX protein expression in the ARX ko clones, we utilized a previously described pancreatic differentiation protocol that generates a mixed population of endocrine cells including glucagon-positive cells which express ARX (Fig 2A) [26]. At day 26 of differentiation, WT cells expressed ARX in both chromogranin A-positive (endocrine) and negative (non-endocrine) populations, resembling the pattern of ARX expression in human fetal pancreas tissue at 13 weeks of gestation (Fig 1D) when ARX is found in cells expressing glucagon with or without insulin (Fig 1E). In contrast, ARX ko clones had no ARX immunoreactivity as predicted by their genotype (Fig 1D).

### Pancreatic Differentiation of ARX ko hESCs

**Fig 3:**
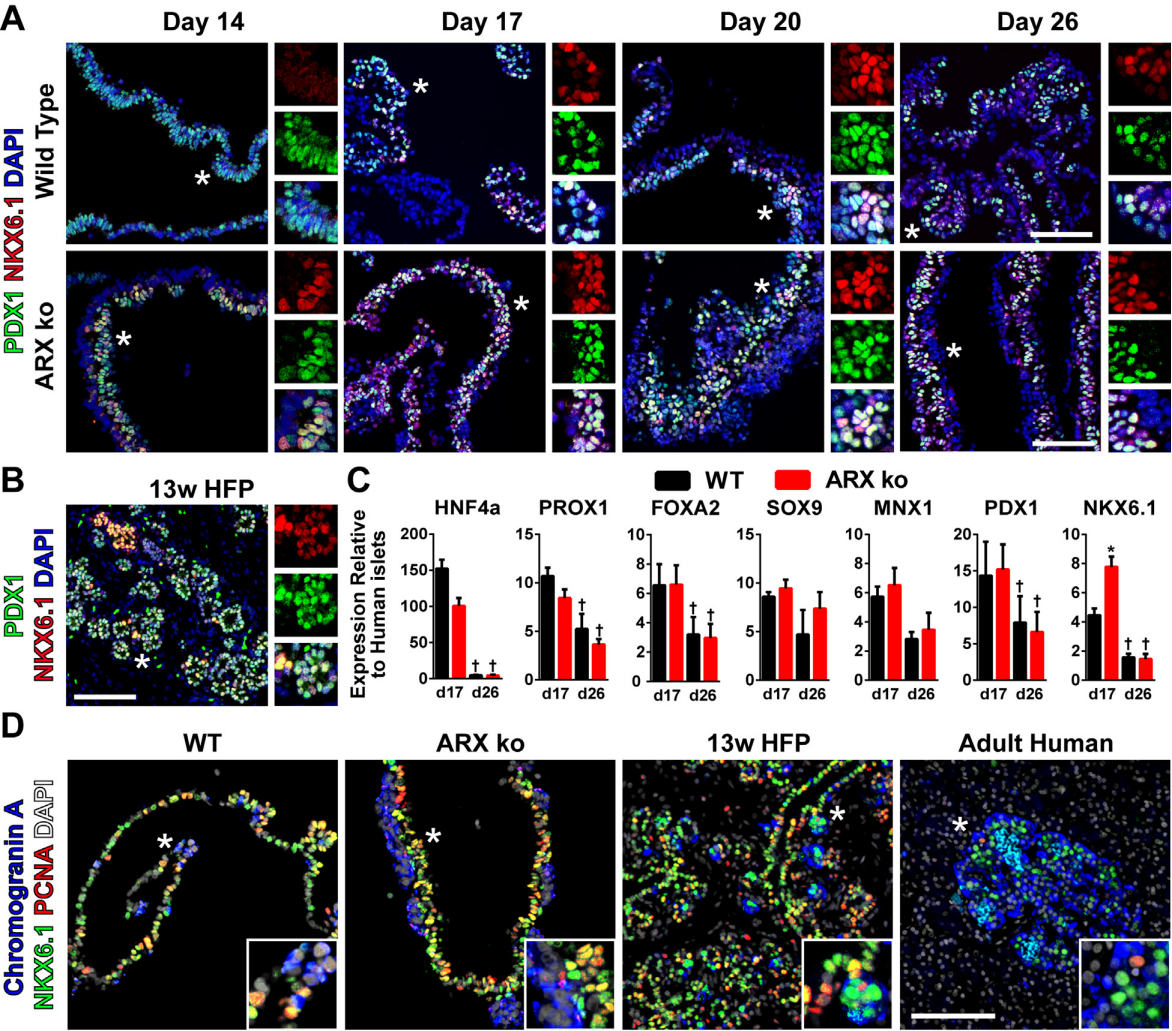
Pancreatic Progenitors in ARX ko hESCs. (A) Immunostaining of PDX1 (green) and NKX6.1 (red) in agarose-embedded wild type (WT) and ARX knockout (ARX ko) cell sheets from day 14, 17, 20, and 26 of culture. * indicates region of enlargement (right) which includes individual red and green channels and overlay with DAPI (blue) showing coexpression patterns. (B) Immunostaining of 13 week human fetal pancreatic tissue the same as A. (C) RT-qPCR of day 17 and day 26 whole population samples examining the expression of transcription factors believed to be involved in pancreatic progenitor induction. Wild type (black bars) and ARX ko clone 1 (red bars) cell samples relative to adult human islets expression levels. † indicates p < 0.05 within the genotype over time, * indicates p < 0.05 wild type vs ARX ko at given culture day, N = 4 per group. (D) Immunostaining of NKX6.1 (green), proliferating cell nuclear antigen (PCNA, red), and chromogranin A (blue) in 26 day differentiated wild type and ARX ko cells, 13 week human fetal pancreatic tissue, and adult human pancreatic tissue. Inset is a ~3x enlarged portion from the region indicated by the star. Scale bar is 100 μm for all panels.

To control for potential off-target genomic editing by the zinc-finger nuclease (ZFN) pair, both ARX ko clones with independent deletions were differentiated in parallel. Similar to WT hESCs, both ARX ko clones efficiently formed CXCR4-positive definitive endoderm cells and PDX1-positive foregut endoderm (Fig 2B). However, ARX ko clones had more NKX6.1-positive pancreatic progenitors at day 17, as measured by flow cytometry (Fig 2B) and RT-qPCR (Fig 3C). ARX ko pancreatic progenitors co-expressed PDX1 and NKX6.1 between days 14 to 26 of differentiation (Fig 3A and 3B) and NKX6.1-positive progenitors were proliferative as determined by PCNA staining similarly to WT and human fetal samples (Fig 3D). Importantly, markers of early pancreatogenesis (*HNF4a*, *PROX1*, *FOXA2*, *PDX1*) decreased between days 17 to 26 in both WT and ARX ko cells. Taken together, these data suggest that ARX ko does not affect germ layer specification or gut tube regionalization *in vitro*, but may affect the formation or specification of NKX6.1-positive progenitors.

As previously described for this differentiation protocol, we expected early immature hormone-positive cells to form after day 17 of culture [26]. ARX ko clones released significantly less glucagon and human C-peptide throughout differentiation under basal conditions and in response to potassium chloride (KCl) at day 24 (Fig 2C and 2E) and had decreased cellular content on day 26 (Fig 2D and 2F). Despite similar levels of basal somatostatin secretion (Fig 2G) and total content (Fig 2H), ARX ko cells released more somatostatin upon KCl stimulation compared to WT cells (Fig 2G). Thus the formation of glucagon and insulin-positive lineages was partially disrupted in ARX ko cells while the somatostatin-positive population released more somatostatin when stimulated.

### Pancreatic Endocrine Specification of ARX ko cells

**Fig 4:**
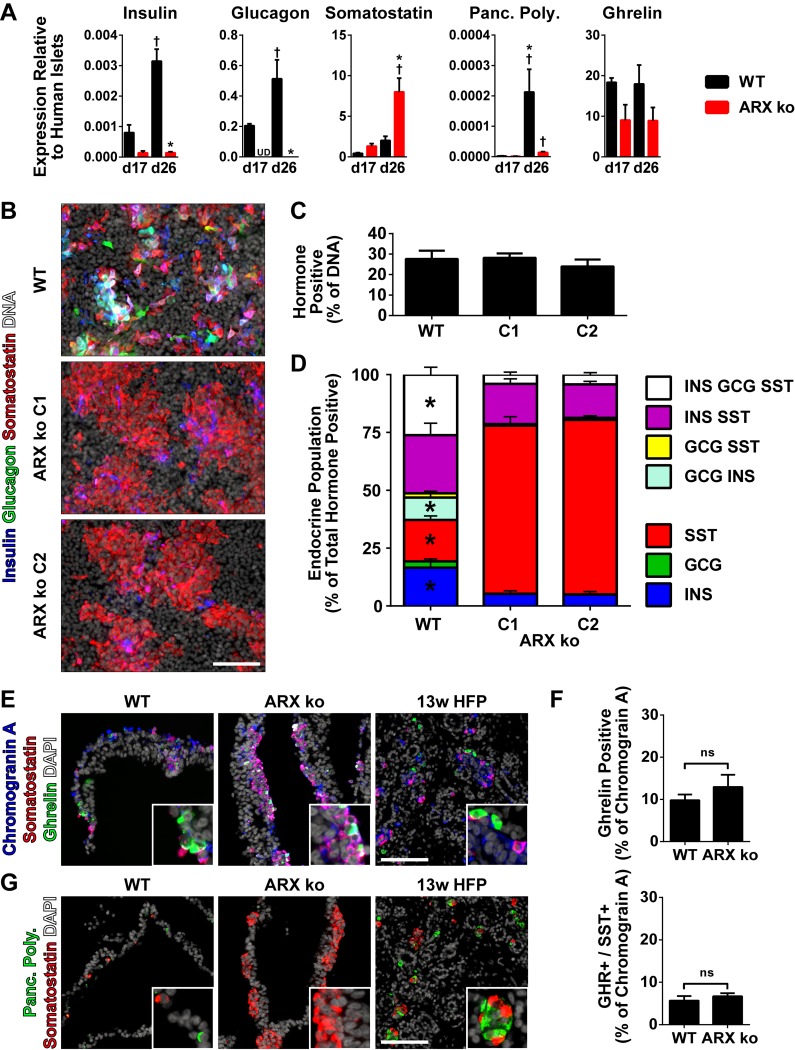
Pancreatic Endocrine Profile of ARX ko hESCs. (A) RT-qPCR of day 17 and day 26 whole population samples examining the expression of the five major pancreatic hormones in wild type (WT; black bars) and ARX knockout clone 1 (ARX ko; red bars) relative to adult human islets expression levels. † indicates p < 0.05 within the genotype over time, * indicates p < 0.05 wild type vs ARX ko at given culture day. (B) 26-day differentiated cultures were stained *in situ* for insulin (blue), glucagon (green), somatostatin (red), and nuclei (white). (C) Image based cell counting was performed to determine the number of cells positive for any of the three hormones as a percentage of the total number of nuclei. (D) Single cell pancreatic endocrine population profile as a percentage of the total number of insulin, glucagon and somatostatin-positive cells. * indicates significant changes in the population between wild type and ARX ko (both clones). (E) Immunostaining of 13w HFP and 26-day differentiated wild type and ARX ko cells for chromogranin A (blue), somatostatin (red), ghrelin (green) and DAPI (white). (F) Single cell quantification of ghrelin-positive and ghrelin/somatostatin co-positive cells as a percentage of the total hormone (chromogranin A) positive fraction. (G) Immunostaining of pancreatic polypeptide-positive cells in day 26 cultures and 13w HFP with pancreatic polypeptide (green), somatostatin (red) and DAPI counterstain (white). Inset is a ~3x enlarged portion from the region indicated by the star. Scale bar is 100 μm for all panels.

Compared to WT cells at day 26, ARX ko cells expressed decreased insulin, glucagon, and PP mRNA, along with increased somatostatin transcript levels with no change in ghrelin (Fig 4A). Immunostaining of 26-day differentiated cells revealed that the total number of endocrine cells (any combination of insulin, glucagon and/or somatostatin) was not changed in ARX ko cells compared to WT cells (Fig 4B and 4C). However, while WT cells contained a mixture of various polyhormonal cells, ARX ko clones were composed primarily of somatostatin-positive cells which were negative for insulin and glucagon and somatostatin/insulin co-positive cells (Fig 4D). Furthermore, within the endocrine cell fraction, the total number of somatostatin-positive cells (regardless of co-expression with other hormones) was higher in ARX ko cells compared to WT cells (WT: 71±4%, ARX ko C1: 94±1% ARX ko C2: 94±1%, p<0.05). ARX ko endocrine cells also had a decrease in the total number of insulin-positive cells (WT: 78±1%, ARX ko C1: 27±4%, ARX ko C2: 24±2%, p<0.05) and glucagon-positive cells (WT: 40±8%, ARX ko C1: 4±1%, ARX ko C2: 5±1%, p<0.05). There was no difference in the expression of ghrelin (Fig 4E and 4F) by immunostaining, but PP was undetectable in ARX ko cells (Fig 4G).

**Fig 5:**
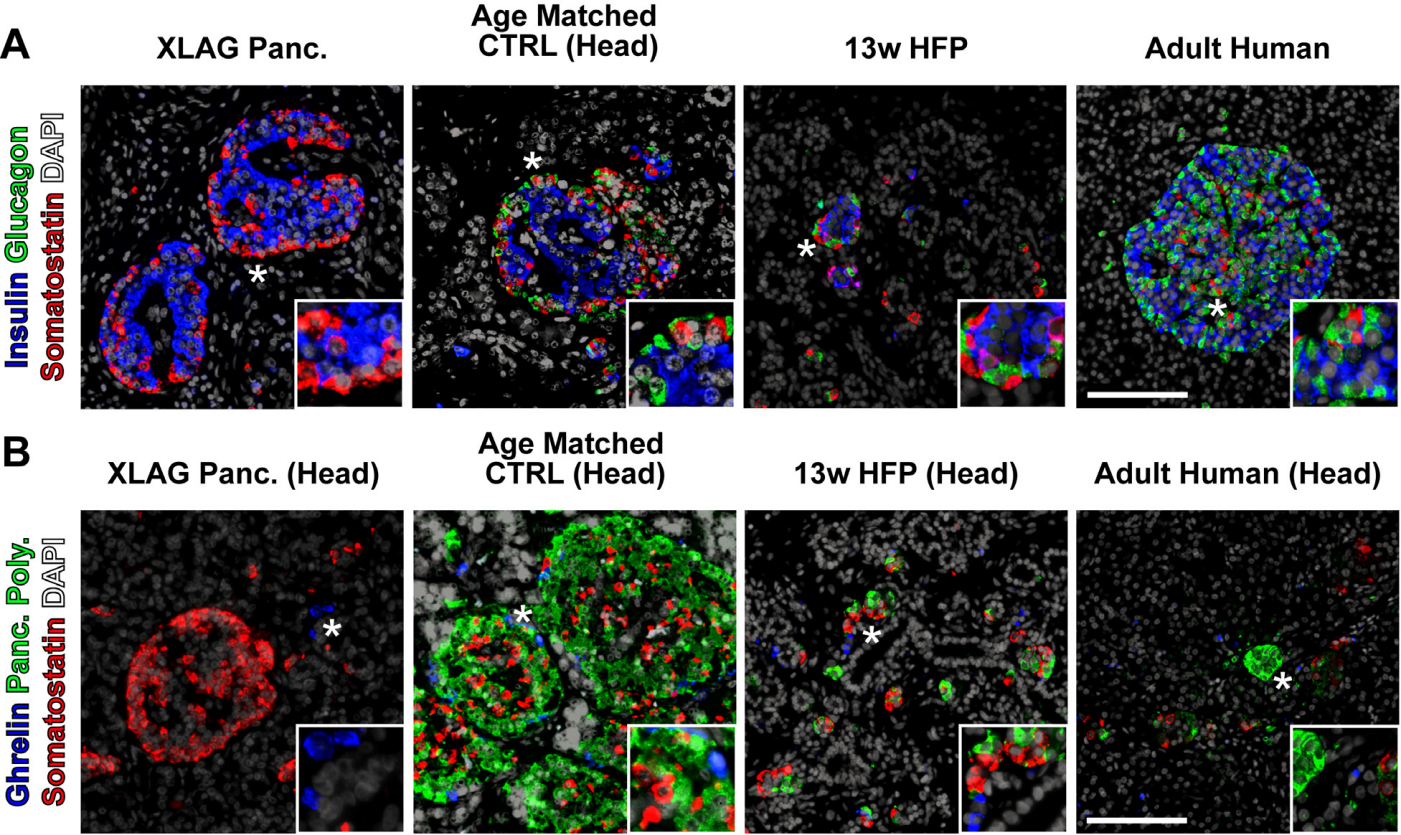
XLAG Pancreatic Endocrine Profile. 16-month old XLAG (ARX knockout) pancreatic tissue, age-matched control pancreatic tissue, 13w human fetal pancreatic tissue, and adult human pancreatic tissue were immunostained for insulin (blue), glucagon (green), somatostatin (red) and DAPI (white) in (A) and ghrelin (blue), pancreatic polypeptide (green), somatostatin (red) and DAPI (white) in (B). "Head" indicates specific sample from head region of the pancreas. Inset is a ~3x enlarged portion from the region indicated by the star. Scale bar is 100 μm for all panels.

For a comparison to our hESC-derived ARX ko endocrine cells, we also examined human pancreatic tissue samples from a 16-month old patient with XLAG (*ARX* deficiency) and controls [17, 30]. Immunostaining of normal pancreatic tissue from a 13 week human fetus, a 16 month old and an adult human revealed the typical arrangement of pancreatic endocrine cells (expressing either insulin, glucagon, somatostatin, PP or ghrelin). In contrast, XLAG pancreatic tissue lacked glucagon and PP-positive cells, but contained insulin, somatostatin, and ghrelin-positive cells (Fig 5). Taken together, the hESC-derived ARX ko cells resembled the human XLAG endocrine pancreas in terms of the glucagon and PP deficiency, but differed in terms of the insulin deficiency in ARX ko clones.

**Fig 6:**
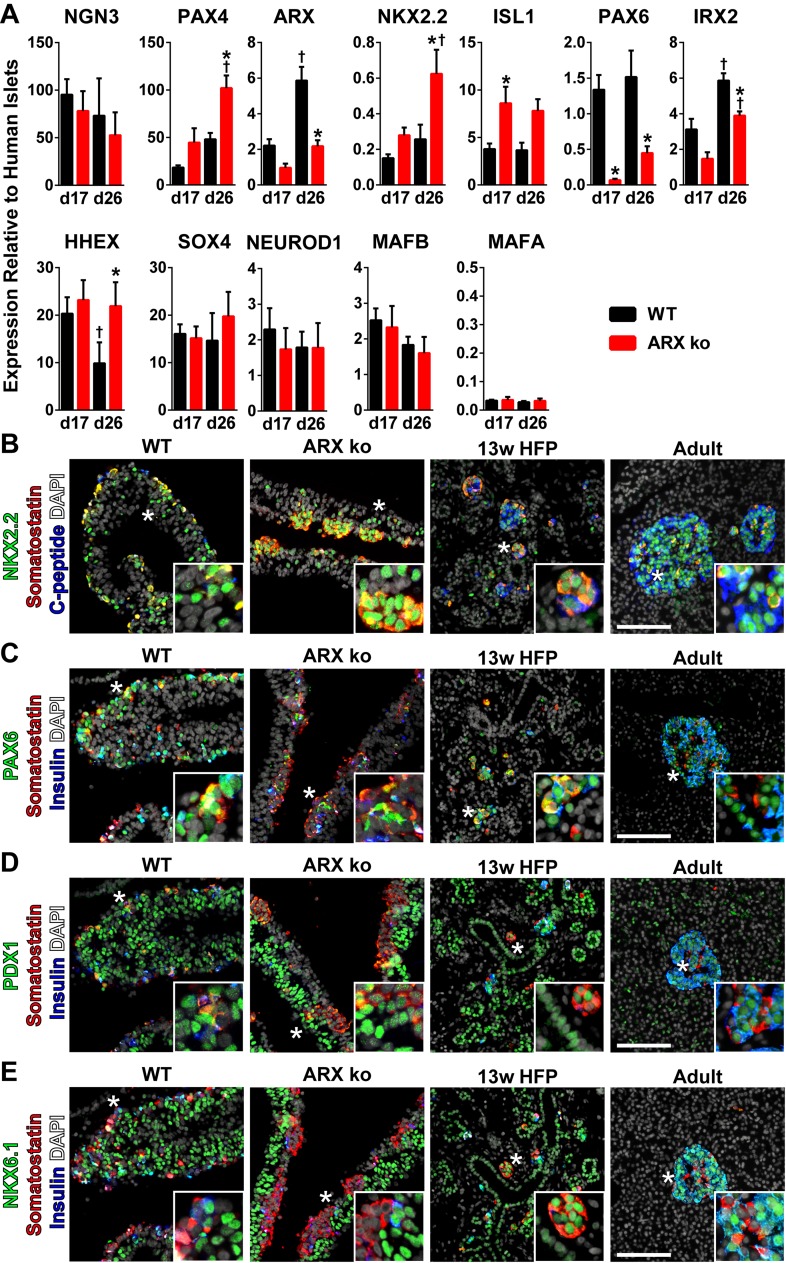
Pancreatic Fate specification in ARX ko hESCs. (A) RT-qPCR of day 17 and day 26 whole population samples examining the expression of transcription factors believed to be involved in pancreatic endocrine fate specification. Wild type (WT; black bars) and ARX knockout clone 1 (ARX ko; red bars) cell samples relative to adult human islets expression levels. † indicates p < 0.05 within the genotype over time, * indicates p < 0.05 wild type vs ARX ko at given culture day, N = 4 per group. (A-E) Immunostaining of 26-day differentiated wild type and ARX ko cells, 13 week human fetal pancreatic tissue and adult human pancreatic tissue. Inset is a ~3x enlarged portion from the region indicated by the star. Scale bar is 100 μm for all panels.

To understand what may be driving the diminished insulin levels in differentiated hESC-derived ARX ko cells, we examined expression of key pancreatic endocrine transcription factors. While *NGN3* levels were unchanged, *PAX4* was elevated in differentiated ARX ko cells, and was correlated with decreased levels of *ARX* mRNA potentially due to PAX4 mediated repression of *ARX* or altered *ARX* mRNA stability (Fig 6A). Expression of *NKX2*.*2*, *ISL1*, and *HHEX* were elevated in differentiated ARX ko cells while *PAX6* and *IRX2* levels were reduced compared to WT cells (Fig 6A). Other transcription factors including *SOX4*, *NEUROD1*, and *MAFB* were unchanged in differentiated cells (Fig 6A). Notably, *MAFA* expression levels were unchanged between genotypes and were relatively low compared to adult human islets (Fig 6A). These *MAFA* levels are reminiscent of immature fetal endocrine expression patterns prior to glucose-responsive secretion capacity as previously reported for CA1S hESCs differentiated using this protocol [26], and would be expected to be increased following recently developed *in vitro* maturation protocols [31].

**Fig 7:**
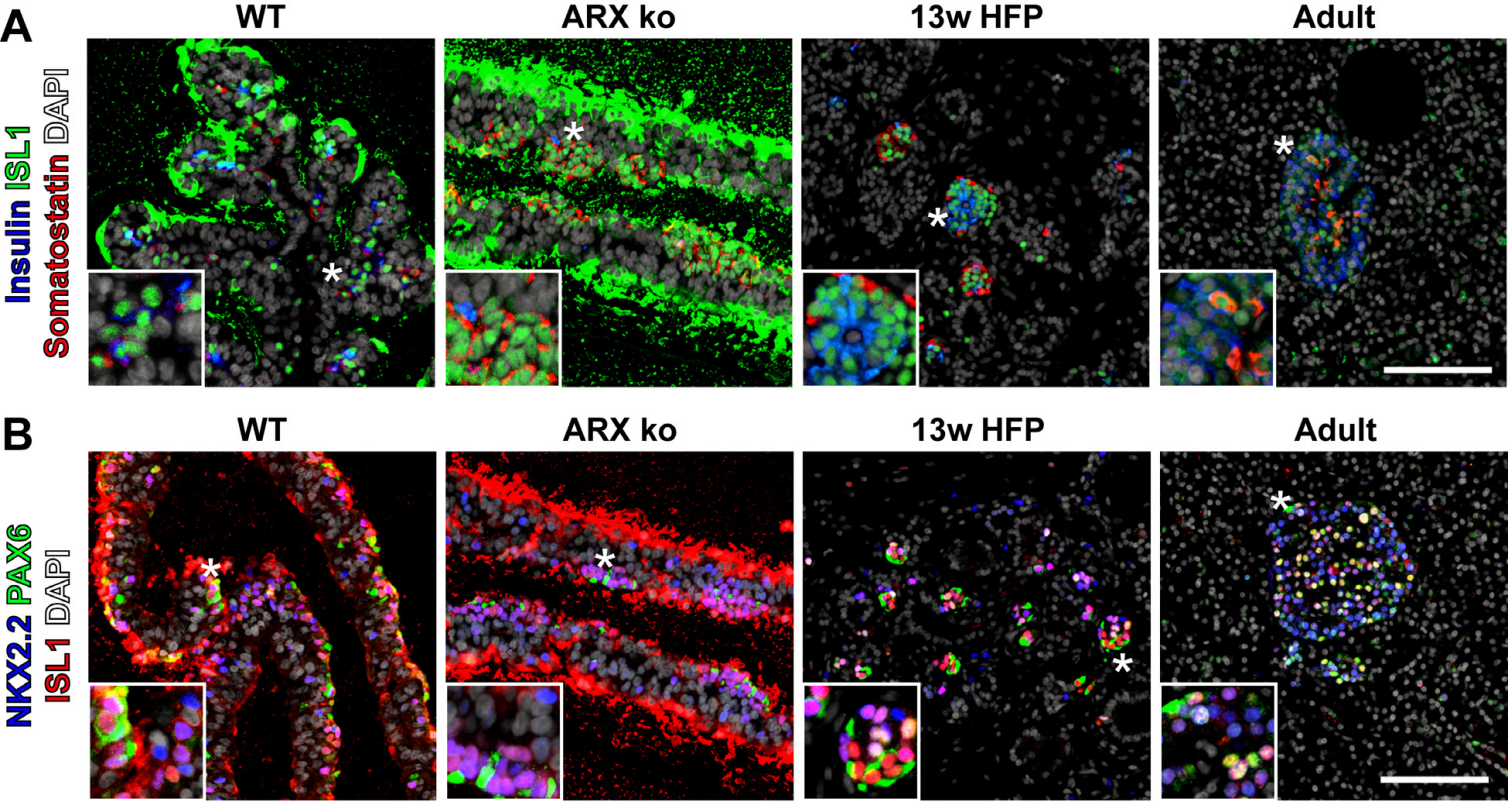
Expression of ISL1 in ARX ko hESCs and Human Tissue. (A) Immunostaining of ISL1 (green), somatostatin (red), and insulin (blue) in 26 day differentiated wild type (WT) and ARX knockout cells (ARX ko), 13 week human fetal pancreatic tissue, and adult human pancreatic tissue. (B) Immunostaining of PAX6 (green), ISL1 (red), and NKX2.2 (blue) in the same sample series as above. Inset is a ~3x enlarged portion from the region indicated by the star. Scale bar is 100 μm for all panels. Extracellular immunoreactivitity of ISL1 is a staining artifact associated with the agarose which surrounds the embedded cell sheets.

**Fig 8:**
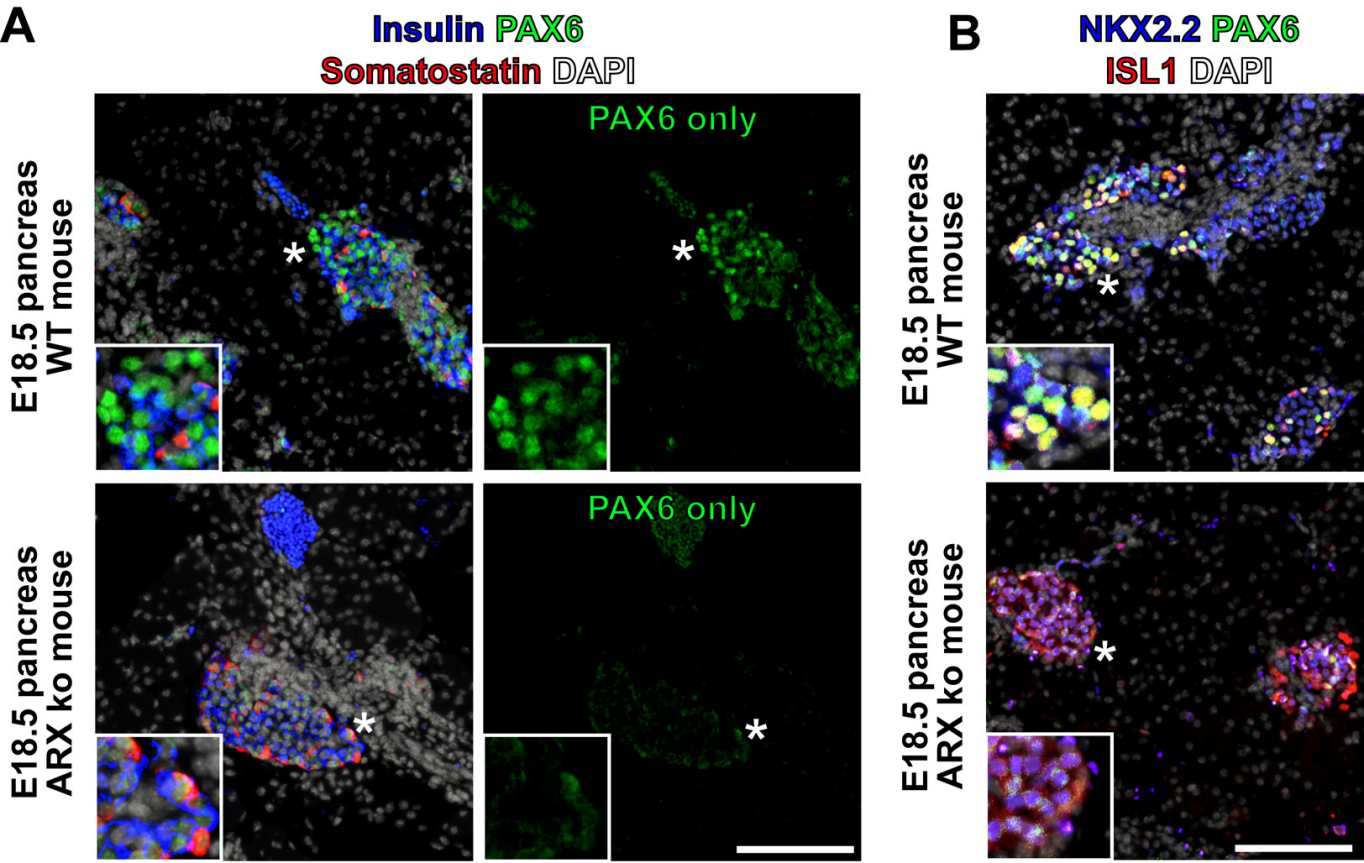
Expression of PAX6 in ARX ko mice. (A) Immunostaining of PAX6 (green), somatostatin (red), and insulin (blue) in ARX knockout (ARX ko) and wild type (WT) E18.5 mouse pancreatic samples. Image on the right shows individual PAX6 channel showing nuclear PAX6 in wild type islet cells and lower levels of PAX6 in ARX ko tissue. (B) Immunostaining of PAX6 (green), ISL1 (red), and NKX2.2 (blue) in the same samples as in A. Inset is a ~3x enlarged portion from the region indicated by the star. Scale bar is 100 μm for all panels.

We next examined the expression patterns of a subset of these transcription factors by immunostaining differentiated ARX ko and WT cells, as well as human fetal and adult pancreatic tissue. Similar to adult, fetal and WT hESC-derived samples, ARX ko somatostatin-positive cells showed prominent nuclear localization of NKX2.2 (Fig 6B) and ISL1 (Fig 7B). PAX6 immunoreactivity was relatively weak in ARX ko cells but robust and nuclear in WT, fetal and adult pancreas samples (Fig 6C). PDX1 and NKX6.1 were observed in hormone-negative cells from WT, ARX ko, and human fetal pancreatic tissues (Fig 6D and 6E). Interestingly, somatostatin-positive cells from the ARX ko hESCs expressed weak nuclear PDX1 and no NKX6.1 similarly to adult δ-cells, whereas somatostatin-positive cells from human fetal pancreas expressed both PDX1 and NKX6.1 (Fig 6D and 6E). Similarly to ARX ko hESC-derived somatostatin-positive cells, E18.5 Arx ko mouse pancreata contained endocrine clusters with nuclear NKX2.2 and ISL1, but weak PAX6 immunoreactivity (Fig 8). In contrast, adult human islets contained nuclear expression patterns of NKX2.2, ISL1 and PAX6 (Fig 7B). Taken together, ARX ko hESCs are biased towards the formation of somatostatin-positive cells, which express no NKX6.1, low PDX1, high NKX2.2, high ISL1, and low/no PAX6. As a similar pattern of transcription factors is observed in δ-cells from ARX ko mice and in a subset of developing human somatostatin-positive cells, this pattern may represent the natural developmental trajectory of δ-cell specification.

**Fig 9:**
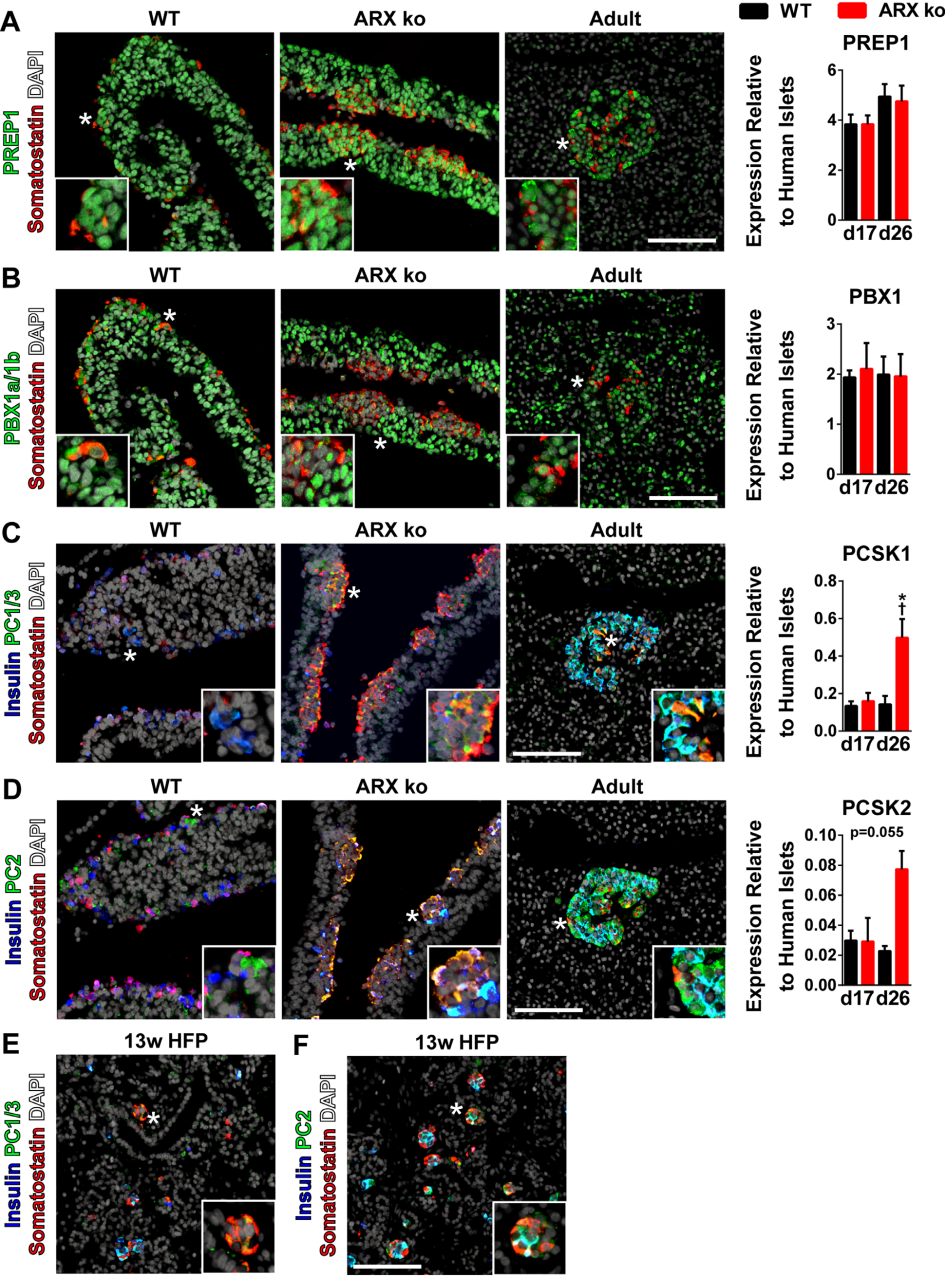
Expression of PREP1, PBX1 and Processing of Somatostatin in ARX ko hESCs. (A—D) Immunostaining of PREP1, PBX1a/1b, PC1/3, PC2 (all green), somatostatin (red), and insulin (blue) in 26 day differentiated wild type (WT) and ARX knockout (ARX ko) cells, and adult human pancreatic tissue. RT-qPCR of day 17 and day 26 whole population samples examining the expression of transcription factors believed to be involved expression and processing of somatostatin. Wild type (WT; black bars) and ARX ko clone 1 (ARX ko; red bars) cell samples relative to adult human islets expression levels. † indicates p < 0.05 within the genotype over time, * indicates p < 0.05 wild type vs ARX ko at given culture day, N = 4 per group. (E and F) Immunostaining of 13 week human fetal pancreatic tissue for expression of PC1/3 and PC2 (both green), somatostatin (red), and insulin (blue). DAPI is white in all images. Inset is a ~3x enlarged portion from the region indicated by the star. Scale bar is 100 μm for all panels.

Since ARX ko endocrine cells developed primarily into KCl-responsive, somatostatin-secreting cells (Fig 2C), we examined expression of factors known to regulate somatostatin transcription, including PDX1, PBX1, and PREP1 [32]. As mentioned, ~95% differentiated cells at day 11 expressed PDX1 (Fig 2B) and PDX1 was highly expressed throughout pancreatic progenitor population between days 14–26 in both wild type and ARX ko cells (Figs 3A and 6D). We also found that both *PREP1* and *PBX1* mRNA levels were 2–5 fold higher than adult human islets in both WT and ARX ko cell samples at days 17 and 26 of differentiation (Fig 9A and 9B). PREP1 and PBX1 immunoreactivity was nuclear and localized to both the pancreatic progenitor and endocrine compartments, including somatostatin-positive cells of WT and ARX ko cell samples in a similar pattern to somatostatin-positive δ-cells in adult human tissue (Fig 9A and 9B). Together these data suggest that PREP1, PBX1 and PDX1 are present in somatostatin-positive cells and may promote somatostatin expression.

The somatostatin pro-protein is processed by a number of prohormone convertases including PC1/3, PC2, furin, PACE4, and PC5 [33]. In the context of the pancreatic δ-cell, the liberation of somatostatin-14 is achieved by activity of PC1/3 and/or PC2 [34, 35]. Differentiated ARX ko cells had elevated levels of *PCSK1* and *PCSK2* compared to WT cells and both PC1/3 and PC2 immunoreactivity was found in somatostatin-positive cells from hESC-derived cells, adult human pancreas and 13 week human fetal pancreas (Fig 9C and 9E). Given the expression levels and distribution patterns of these processing enzymes, it is likely that the WT and ARX ko somatostatin-positive cells generate mature pancreatic somatostatin-14.

### Re-expression of ARX Restores Insulin Expression in ARX ko cells

**Fig 10:**
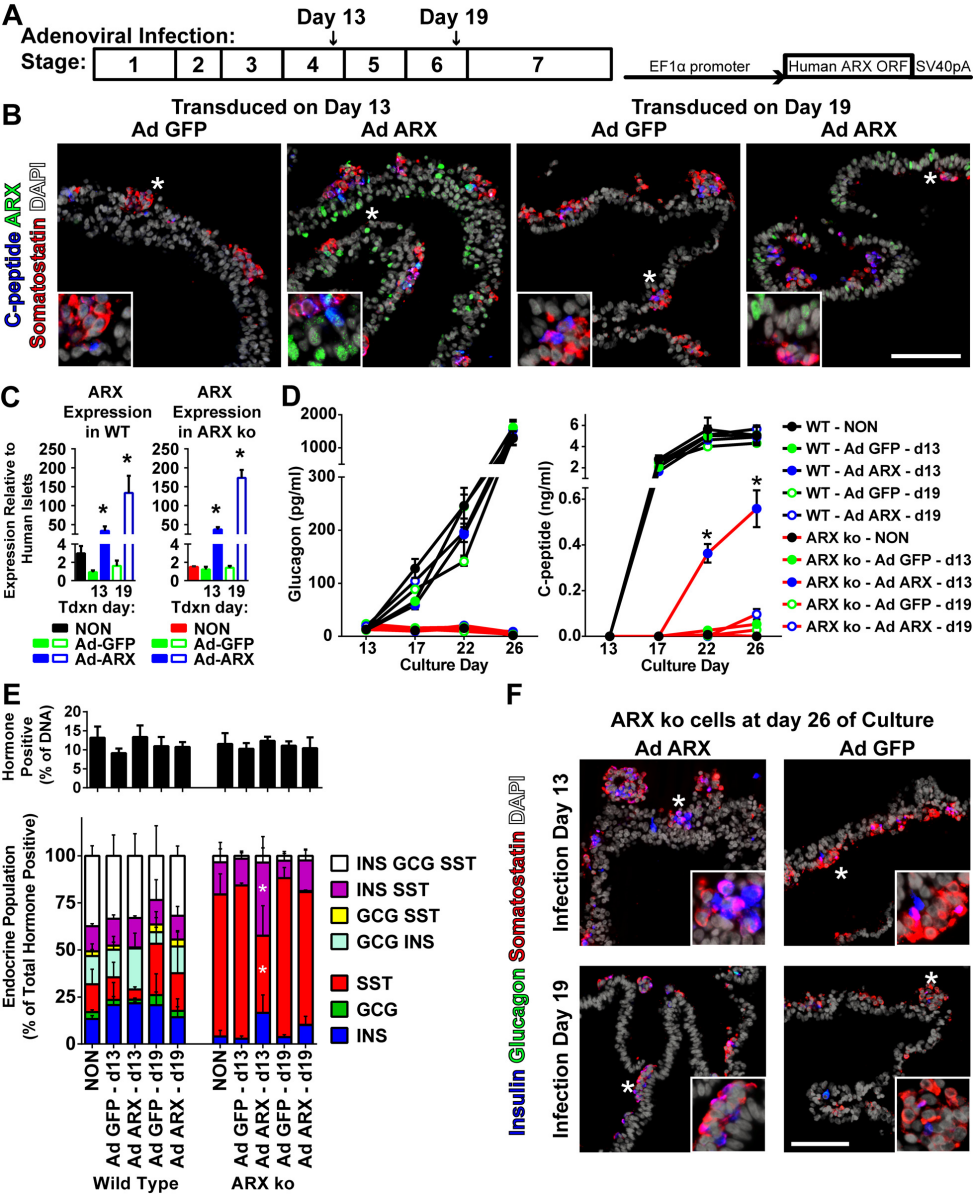
Adenoviral ARX Expression in Developing ARX ko hESCs. (A) Schematic diagrams of adenoviral infection timeline during pancreatic differentiation stages and the adenoviral construct used to express human ARX under the constitutive EF1α promoter. (B) Immunostaining of ARX (green), somatostatin (red), and C-peptide (blue) in ARX knockout (ARX ko) cells differentiated to day 26 after transduction at day 13 or 19 with Ad GFP or Ad ARX. (C) RT-qPCR of day 26 whole population samples examining the expression of ARX in wild type (WT) (left graph) and ARX ko clone 1 (right graph) cells treated with Ad GFP (green bars) or Ad ARX (blue bars) at an MOI of 2 on day 13 (filled bars) or 19 (open bars). * indicates p < 0.05 Ad GFP vs Ad ARX from a given transduction (Tdxn) day. (D) Glucagon and C-peptide levels from 24-hour static media samples taken between days 13 and 26 comparing Ad ARX treatment in wild type cells (black lines) and ARX ko cells (red lines). Treatments are indicated by symbol colour in the figure. * indicates p < 0.05 Ad ARX on day 13 versus all other populations on day 22 and 26. (E—F) Immunostaining and single cell hormone analysis of Ad GFP and Ad ARX treated wild type and ARX ko day 26 cultures for insulin (blue), glucagon (green), and somatostatin (red). (E) Total number of hormone-positive cells (any combination of insulin, glucagon and or somatostatin) represented as a percentage of the total number of nuclei. Endocrine population breakdown of wild type and ARX ko cultures. * indicates p < 0.05 Ad ARX delivered on day 13 versus no virus (NON) and Ad GFP delivered on day 13 in ARX ko cells. N = 3 per group. Inset is a ~3x enlarged portion from the region indicated by the star. Scale bar is 100 μm for all panels.

**Fig 11:**
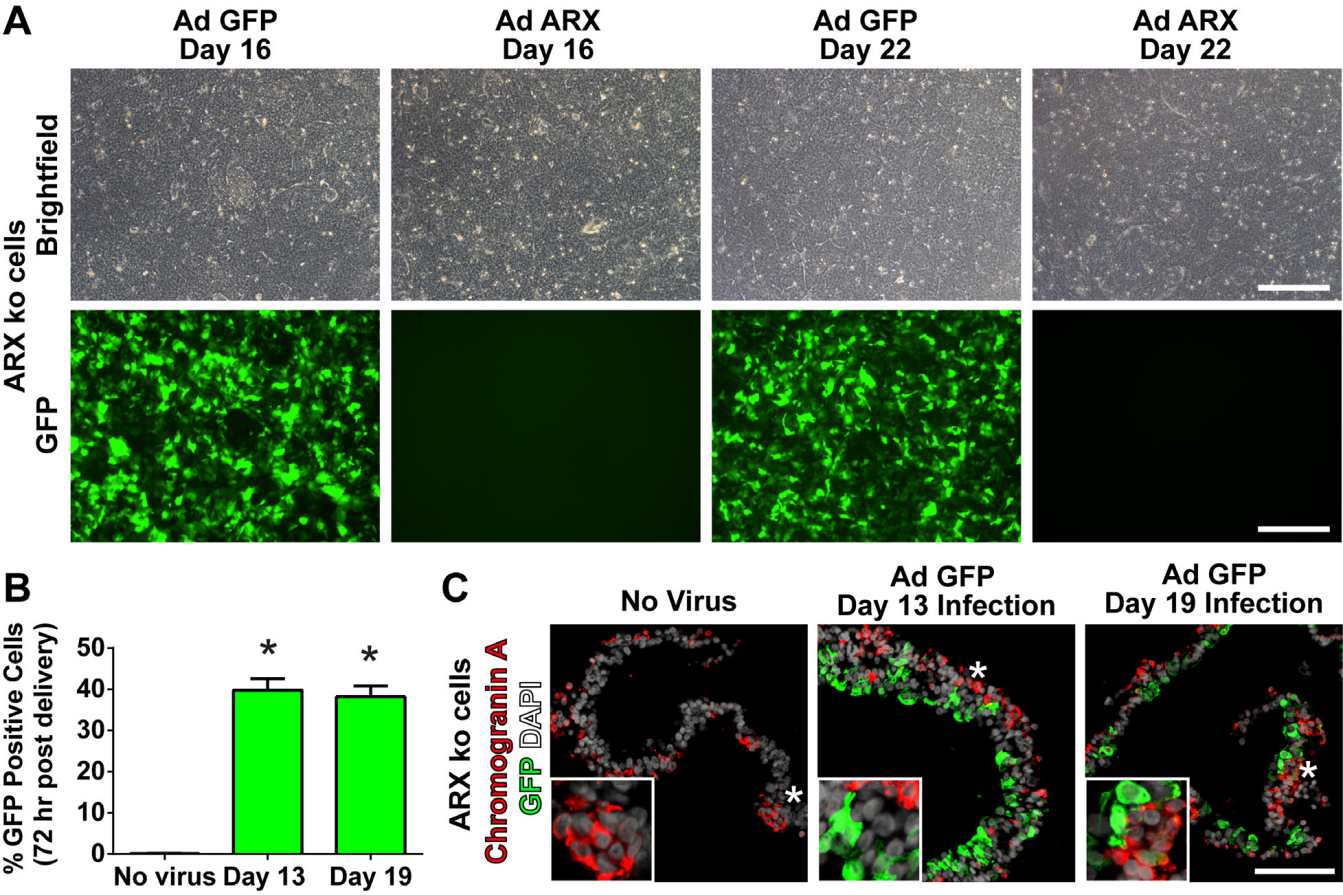
Adenoviral Infection Efficiency in ARX ko Cells. (A) Brightfield and green channel (GFP) images of differentiating ARX knockout (ARX ko) cell cultures 72 hours post adenoviral infection at a MOI of 2. (B) GFP infection efficiency quantified by flow cytometry 72 hours after viral delivery as a percentage of the total population. (C) Transgene expression (GFP) in 26-day differentiated ARX ko hESCs based on immunostatining of GFP (green) and chromogranin A (red). Nuclei are counterstained with DAPI (white). Inset in C is a ~3x enlarged portion from the region indicated by the star. Scale bar is 200 μm for panel A and 100 μm for C.

**Fig 12:**
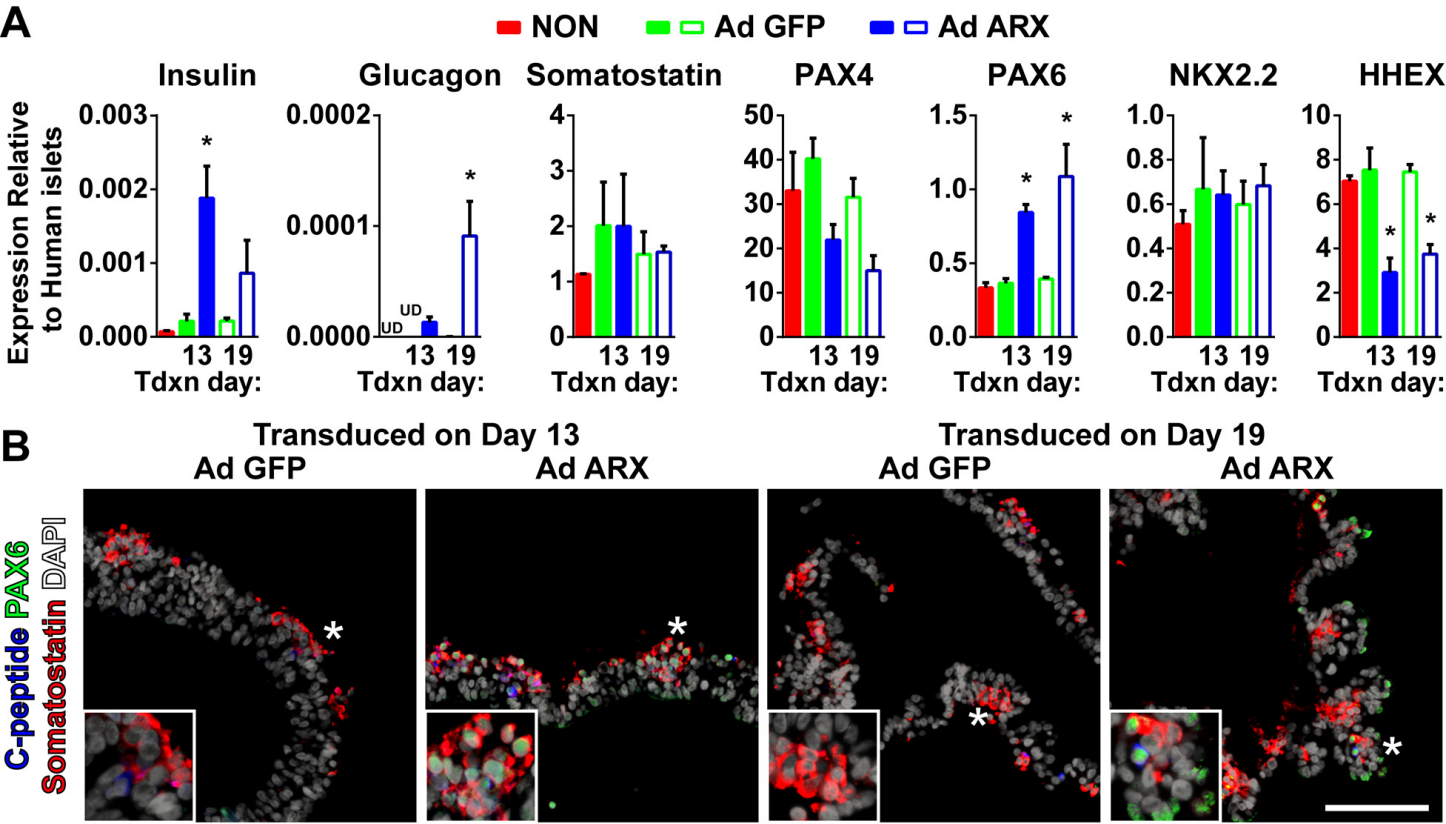
Fate Specification Factors in Ad ARX Treated ARX ko hESCs. (A) RT-qPCR of day 26 whole population samples examining the expression of three hormones and key transcription factors in ARX ko clone 1 cells treated with Ad GFP (green bars) or Ad ARX (blue bars) at an MOI of 2 on day 13 (filled bars) or 19 (open bars). * indicates p < 0.05 Ad GFP vs Ad ARX from a given transduction (Tdxn) day. N = 3 per group. (B) Immunostaining of PAX6 (green), somatostatin (red), and C-peptide (blue) in ARX ko cells differentiated to day 26 after transduction at day 13 or 19 with Ad GFP or Ad ARX. Inset is a ~3x enlarged portion from the region indicated by the star. Scale bar is 100 μm for all panels.

Given that differentiated ARX ko hESCs failed to generate glucagon-positive cells and produced minimal insulin-positive cells, we examined whether this phenotype could be rescued by re-expression of *ARX* at specific developmental time points. To do this, we generated an adenoviral vector that permitted expression of the human *ARX* open reading frame under control of the EF1α promoter (Ad ARX). Using a low multiplicity of infection (MOI of 2) early pancreatic progenitors and pancreatic endocrine cells were targeted at days 13 and 19 of culture respectively (Fig 10A). At 72 hours post infection, gene delivery approached 40% of the cell population and was maintained until the end of the differentiation protocol (Fig 11). Re-expression of ARX was similar in ARX ko cells treated at either day 13 or 19, with immunoreactivity observed predominantly in hormone negative cell populations (Fig 10B) at total expression levels 50–150 fold higher than adult human islets (Fig 10C). Delivery of Ad ARX on day 13 of culture, but not day 19, was associated with significantly greater release of C-peptide but not glucagon into the culture media compared to ARX ko cells treated with control virus (Ad eGFP) (Fig 10D). While no effect was observed on the total number of hormone positive cells by ARX re-expression, (Fig 10E), ARX ko cells treated with Ad ARX at day 13, but not day 19, had decreased somatostatin-positive cells and increased somatostatin/insulin cells compared to untreated and Ad GFP treated cultures (Figs 10E and 10F and S1). Similarly, re-expression of *ARX* in ARX ko cells increased insulin and *PAX6* transcript levels with a small increase in glucagon, reduction in *HHEX* and no change in somatostatin (Fig 12A). This increase in *PAX6* was also seen at the protein level in ARX ko cells where nuclear PAX6 immunoreactivity was only observed in cells treated with the ARX expression construct (Fig 12B). Taken together, these data indicate that re-expression of ARX in differentiating ARX ko progenitor cells at day 13 of culture rescues WT PAX6 and HHEX expression patterns associated with an approximate doubling of the number of insulin-positive cells within the endocrine population of the final cultures (ARX ko + Ad GFP: 19±1%, ARX ko + Ad ARX: 59±9%, WT: 79±6%).

## Discussion

**Fig 13:**
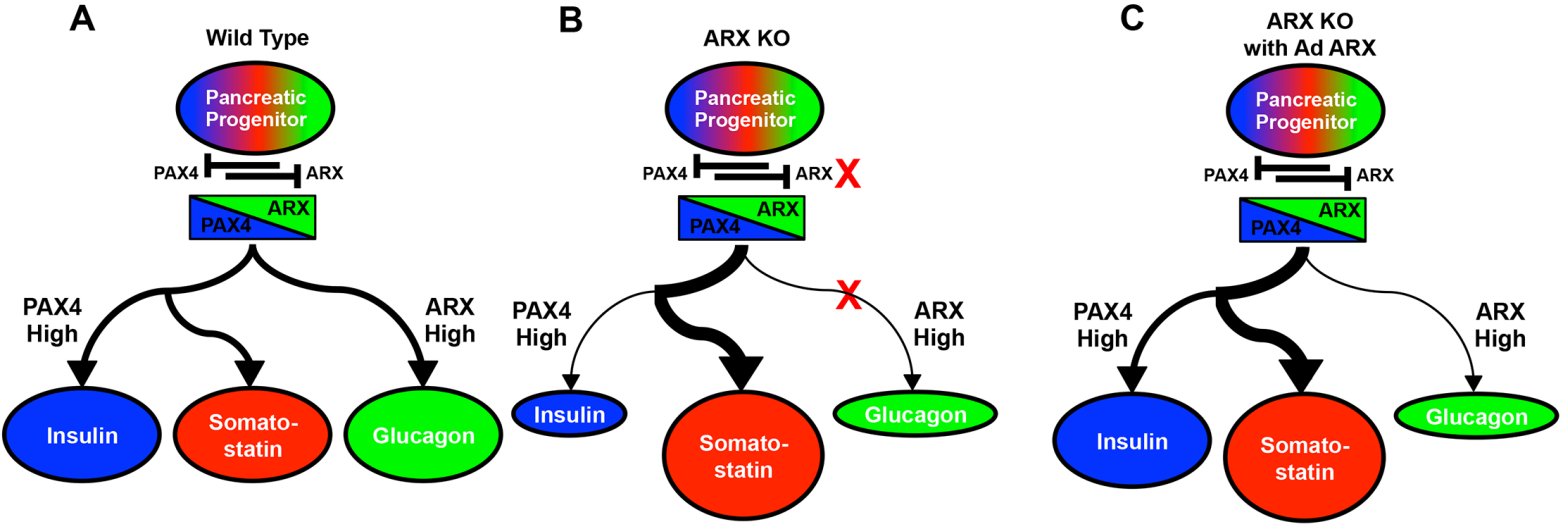
The Role of ARX in Pancreatic Endocrine Specificatin of hESCs. NGN3 induction in hESC derived pancreatic progenitors (PDX+ NKX6.1+) initiates the endocrine lineage. (A) In wild type hESCs, pancreatic endocrine progenitors express both PAX4 and ARX that are mutually corepressive. High expression of ARX specifies and maintains glucagon positive cells while elevated PAX4 levels drives the insulin and somatostatin lineages in normal abundance. (B) In the absence of functional ARX (ARX ko), repression of PAX4 is disrupted leading to a bias toward the insulin/somatostatin lineage. Elevated expression of HHEX as well as altered expression of other transcription factors, leads to large numbers of somatostatin cells at the expense of both insulin and glucagon lineages. (C) Re-expression of ARX in ARX ko pancreatic progenitors temporarily rescues ARX expression resulting in recovery of PAX6 expression (and other factors) which restores the specification of insulin positive cells. Glucagon positive cells are not rescued due to poorly sustained ARX expression in subsequent endocrine cells.

The goal of this study was to examine the role of *ARX* in the context of *in vitro* pancreatic endocrine differentiation of hESCs as a model of human pancreatic development. To do this, we generated small, targeted genetic deletions in the *ARX* locus, isolated clonal cell populations of hESCs with independent null alleles, and subjected them to an established pancreatic differentiation protocol [26]. Although we cannot exclude the possibility of identical phenotype-altering off-target ZFN modifications in both ARX ko clones, it is an unlikely outcome of this genetic modification strategy. Similarly to human pancreatic tissue from a patient with ARX deficiency, ARX ko hESCs generate few if any glucagon and PP-positive cells. Furthermore and in contrast to an ARX deficient human pancreas sample, ARX ko hESCs had decreased formation of insulin-positive cells leaving large populations of somatostatin-positive cells. With re-expression of ARX in ARX ko pancreatic progenitors, the number of insulin-positive cells was restored to near WT levels while the glucagon lineage was not rescued. A model of human pancreatic endocrine specification of WT, ARX ko, and ARX ko with ARX re-expression is summarized in Fig 13. While the ARX ko endocrine phenotype emphasizes a role of ARX in both insulin and glucagon lineages, the incomplete rescue of this phenotype by re-expression of ARX suggests that variation in ARX expression level, timing or target population may distinguish the two lineages. To provide a developmental comparison of human somatostatin-positive cell formation, human adult and fetal pancreatic tissue were characterized for expression of a number of candidate δ-cell transcription factors. This examination along with comparisons between differentiated WT and ARX ko cells suggests a potential role of PAX6 and HHEX in the specification of insulin, glucagon, and somatostatin lineages.

ARX ko clones were similar to WT cells in terms of pluripotency marker expression and differentiation to definitive endoderm and foregut endoderm, supporting the targeted nature of the *ARX* deletions, and the minimal role of ARX in these developmental stages. Since ARX ko pancreatic progenitors unexpectedly generated reduced numbers of insulin-positive cells compared to WT progenitors, we examined the expression of transcription factors that could contribute to this altered hormonal phenotype.

In mice, PAX4 enhances the formation of both β- and δ-cells [36] and stimulates conversion toward the β-cell lineage when constitutively overexpressed in PDX1-, PAX6-, or glucagon-positive cells during pancreatic development [37]. In *Arx/Pax4* double knockout mice, δ- and PP-cell hyperplasia is observed along with a 90% reduction in α- and β-cells [20]. Additionally, in ESC cultures, overexpression of PAX4 enhances the formation of insulin-positive cells by repressing ARX and glucagon [28, 38, 39]. In our cultures, functional deletion of *ARX* was associated with a 2-fold increase in *PAX4* levels compared to WT, and a 100-fold increase compared to adult human islet levels, consistent with derepression of *PAX4* by ARX. In addition, upon re-expression of ARX by adenovirus, *PAX4* expression tended to be decreased in hESCs, supporting the reciprocal regulatory activity between ARX and PAX4 that has been previously observed in mice [20]. As *PAX4* levels in ARX ko cells were elevated above WT levels (which produce insulin) we reason that PAX4 insufficiency was unlikely to explain the insulin deficiency in the ARX ko cells.

The transcription factor NKX2.2 is a known regulator of pancreatic endocrine cells and a possible candidate involved in the ARX ko insulin deficiency. Mice with a null mutation in *Nkx2*.*2* have reduced numbers of β-, α- and PP-cells, an increase in ghrelin-positive ε-cells and no change in δ-cells [40, 41]. Furthermore, mice lacking both *Nkx2*.*2* and *Arx* have an expansion of ghrelin-positive cells that co-express somatostatin [42]. In our ARX ko cultures, we observed elevated levels of *NKX2*.*2* compared to WT cells and as expected, NKX2.2 was localized predominantly to endocrine cells, including somatostatin-positive cells. However, we observed no increase in ghrelin transcript levels, total ghrelin-positive cell number, or ghrelin / somatostatin co-positive cell number in ARX ko cultures, suggesting that in our system, NKX2.2 levels were sufficiently elevated to block increases in ghrelin-positive cells. In patients with XLAG (*ARX* deficiency), Itoh et al. reported nuclear immunoreactivity of NKX2.2 suggesting that expression of this factor was not dependant on functional ARX expression [17]. Therefore, our *in vitro* model is consistent with human ARX ko patients and confirms that NKX2.2 is unlikely to be causal to the insulin deficiency observed in our ARX ko endocrine cells.

Among the factors examined in this study, PAX6 was the most highly affected pancreatic transcription factor preceding robust hormone induction in differentiated ARX ko cells. In *Pax6* mutant mice, decreased expression of insulin, glucagon, and somatostatin was observed suggesting a critical role of Pax6 in the expression of these hormones or the genesis of the cell types [43, 44]. We found that similar to E18.5 *Arx* ko pancreatic islets, ARX ko cells had decreased *PAX6* levels (>3 fold vs WT) throughout differentiation and were lacking the nuclear PAX6 expression patterns observed in WT hESCs, fetal and adult human pancreas samples. When ARX was re-expressed to ~50 fold adult levels in ARX ko progenitor cells we found elevated levels of nuclear PAX6 that correlated with the return of insulin expression to the ARX ko cultures but only a small increase in glucagon transcript levels. While speculative, this failure to rescue glucagon levels may be due to technical limitations of adenoviral gene delivery to cell cultures with multiple cell layers. This method, while efficient at gene delivery to apical progenitor populations was less efficient at targeting underlying cells and resulted in poorly sustained ARX re-expression in developing endocrine cells. Indeed we noted robust ARX or GFP expression in the surface progenitor population but not in the underlying endocrine cells. If sustained ARX re-expression had been achieved through to the endocrine compartment, we presume that the glucagon lineage would have been rescued. We hypothesize there is a short-term requirement of ARX expression within pancreatic progenitors to specify insulin positive cells but sustained ARX expression is required to specify or maintain glucagon positive cells. Together, these data suggest that PAX6, or other transcription factors which correlate with PAX6 expression such as ARX and potentially other factors, may be limiting transcriptional activator(s) of insulin in developing ARX ko hESCs.

One recently identified regulator of β- and δ-cell specification is MNX1. This transcription factor, well known for its role in many tissues including the pancreas [45–48], stabilizes and maintains early β-cell development and later functional maintenance as embryonic deletion of MNX1 in NGN3cre, PAX6cre and RIP2cre lineages resulted in loss of insulin positive cells and a corresponding increase in somatostatin positive cells [48]. Pan et al. suggest that in mouse development the δ-cell hyperplasia in MNX1 conditional knockout islets was due to a lineage reallocation process where progenitors that normally generate β-cells are directed to the δ-cell lineage. In our hESC culture model we did not observe differences in MNX1 expression between WT and ARX ko cell populations at progenitor or later endocrine stages. However it is possible that the expression level of MNX1 in endocrine progenitors (NGN3+ or PAX6+) was insufficient to repress the elevated *HHEX* levels we observed and efficiently drive specification of insulin positive cells in a manner similar to that recently observed in murine pancreatic development [48].

With respect to the potential functional capacity of the ARX ko endocrine cells, ARX ko somatostatin cells expressed transcription factors known to be expressed in adult pancreatic δ-cells, including NKX2.2 and ISL1, and known regulators of somatostatin expression including PDX1, PBX1 and PREP1 [32] and mature hormone processing including PC1/3 and PC2 [34, 35]. Interestingly, ARX ko cells also had increased expression of *HHEX* which was recently characterized as being required for the formation and function of pancreatic δ-cells [49] and has been observed in hESC-derived somatostatin-positive cells after functional *in vivo* maturation of pancreatic progenitors [50]. These data, along with the elevated KCl-induced somatostatin release from ARX ko cultures, suggest that the endocrine cells may have some functional capacity, although additional characterization is still required.

The difference in endocrine cell populations between the XLAG sample used in this study and ARX ko hESC-derived cells may be due to molecular differences in the *ARX* mutations. While the ARX protein of the ARX ko hESCs has only an octapeptide repressor domain, the ARX protein from the XLAG mutation assessed in this study retains an octapeptide domain, two nuclear localization signals, three poly-alanine repeat segments and an acidic residue containing domain [17, 30], (Fig 1A). Functional studies of ARX have suggested that the multiple nuclear localization signals alter importin 13 mediated ARX localization [51], the acidic domain has been implicated in coordination of DNA binding [52] and the N-terminal octapeptide domain is involved in recruitment of the GROUCHO/TLE1 co-repressor complex [53]. It is conceivable that the XLAG mutation examined in this study may retain functional repressive capacity either by active nuclear localization of the octapeptide domain or through functions of the acidic domain that is critical to allowing induction and maintenance of insulin positive cells. As the ARX ko mutations in our hESCs are more complete and thus lack these additional components, the generated octapeptide domain, if not degraded, may be unable to access the nucleus to perform its repressive functions. As GROUCHO/TLE1 has be previously implicated in the regulation of α and β-cell identity [54], further insights regarding the interaction between ARX and GROUCHO/TLE1 may yield insights into how ARX regulates pancreatic endocrine specification of hESCs.

## Conclusions

In summary, we have generated and examined novel hESC populations that lack functional ARX expression. These ARX ko hESCs have impaired development of insulin, glucagon and PP expressing lineages and favour development of somatostatin-positive cells with similarities to both human fetal and adult δ-cells. These studies highlight the importance of understanding the roles that transcription factors play during *in vitro* hESC differentiation in order to better model human development and ultimately control the formation of specific pancreatic endocrine cells from hESCs, while also using this system to explore the mechanisms of human diseases of pancreas development.

## Supporting Information

S1 Fig Total Hormone Fractions in Ad ARX treated Cultures. Quantification of the total numbers of cells positive for each of the three hormones (insulin, glucagon, and somatostatin) regardless of copositivity graphed as a percentage of the total number cells positive of any of the three hormones. Based on the data from Fig 6. * indicates p < 0.05 Ad ARX delivered on day 13 versus all other ARX knockout (ARX ko) cell samples. N = 3 per group.
(TIF)

S1 Table RT-qPCR Primers. Primers, product sizes and applicable references for transcripts tested in this study.
(PDF)

S2 Table Antibody Sources and Conditions for Immunocytochemistry. Antibody concentrations, sources, and relevant staining conditions as applicable for this study.
(PDF)
