# Supplementary figures and images for: The Role of ARX in Human Pancreatic Endocrine Specification
Source: PLoS One. 2015 Dec 3;10(12):e0144100. doi: 10.1371/journal.pone.0144100 (PMC4669132; doi:10.1371/journal.pone.0144100)

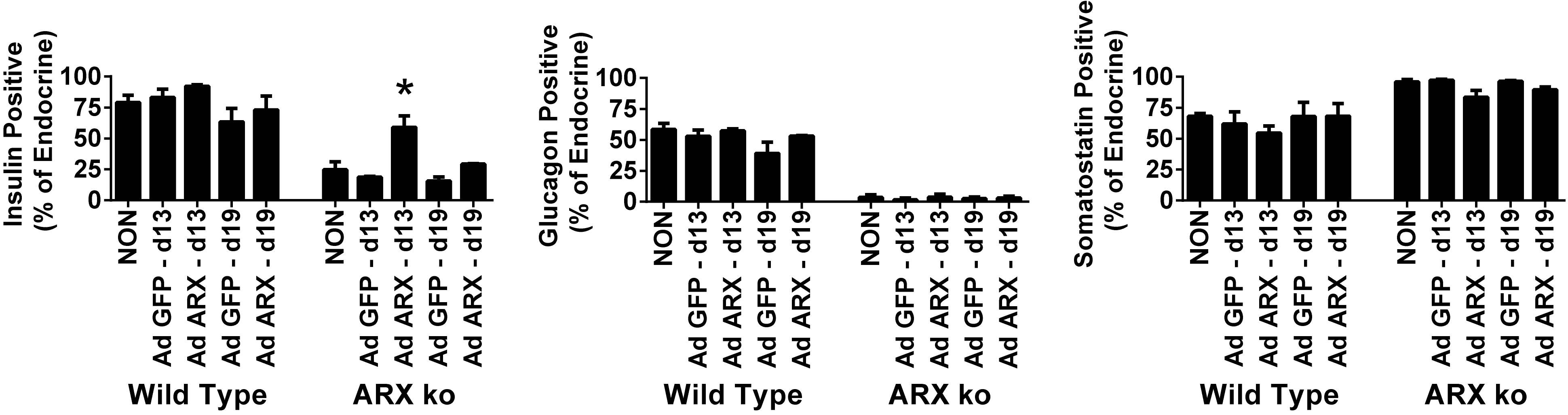

Supplement: S1 Fig — Quantification of the total numbers of cells positive for each of the three hormones (insulin, glucagon, and somatostatin) regardless of copositivity graphed as a percentage of the total number cells positive of any of the three hormones. Based on the data from Fig 6. * indicates p < 0.05 Ad ARX delivered on day 13 versus all other ARX knockout (ARX ko) cell samples. N = 3 per group. (TIF) [file pone.0144100.s001.tif]
