# Supplementary material for: The Role of ARX in Human Pancreatic Endocrine Specification
Source: PLoS One. 2015 Dec 3;10(12):e0144100. doi: 10.1371/journal.pone.0144100 (PMC4669132; doi:10.1371/journal.pone.0144100)
Supplement: S1 Table — Primers, product sizes and applicable references for transcripts tested in this study. (PDF) [file pone.0144100.s002.pdf]

S1 Table. RT-qPCR primers

Gene Name	Gene Accession	Product Size (bp)	Primer Sequence Forward / Reverse 5'→3'	Reference
HNF4a	NM_000457.4	212	TTGCCAACACAATGCCCACT<br>GATAACTTCCTGCTTGGTGATGGTCG	
PROX1	NM_001270616.1	159	GTACGCACGTCAAGCCATCA<br>CGTAATGTGATCTGAGCAACTTCCAG	
FOXA2	NM_021784.4	89	GGGAGCGGTGAAGATGGA<br>TCATGTTGCTCACGGAGGAGTA	[1]
SOX9	NM_000346.3	198	CCAGAATTCCCTTTGGACATTTGTG<br>CTGCTCCATTTAGCCAAGGTTG	
MNX1	NM_005515.3	115	TCGCTCATGCTCACCGAGA<br>CCTTCTGTTTCTCCGCTTCCT	[2]
PDX1	NM_000209	178	CGTCCAGCTGCCTTTCCCAT<br>CCGTGAGATGTACTTGTTGAATAGGA	[3]
NKX6.1	NM_006168	186	GCCCGCCCTGGAGGGACGCA<br>ACGAATAGGCCAAACGAGCCC	[3]
NGN3	NM_020999	286	AGACGACGCGAAGCTCACC<br>AAGCCAGACTGCCTGGGCT	[2]
PAX4	NM_006193	169	AGCAGAGGCACTGGAGAAAGAGTT<br>CAGCTGCATTTCCCACTTGAGCTT	[3]
ARX	NM_139058.2	141	CTGCTGAAACGCAAACAGAGGC<br>CTCGGTCAAGTCCAGCCTCATG	[2]
NKX2.2	NM_002509	221	CTTCTACGACAGCAGCGACAACCCG<br>CCTTGGAGAAAAGCACTCGCCGCTTT	[3]
ISL1	NM_002202	200	GAGCAGCGGCTCTTTCAGC<br>CCGCAACCAACACATAGGGAAATCAG	
PAX6	NM_000280	130	AACCAATTCCACAACCCACCACAC<br>TTATTTGCCATGGTGAAGCTGGGC	
IRX2	NM_033267.4	153	CGGCTACGAGCCCAAGAAAG<br>GCAAGTTGGTGCTGGGAGG	
HHEX	NM_002729.4	198	CATGTTCAGAAAACTGGATTTAGGAATAATGT<br>CCTAAGAGCAGTACATAAACTATTTGTTAAGTC	
SOX4	NM_003107.2	159	CGTTCTCGTCGTCGGATCAA<br>CAACAACATCAATAACAACAATCAACAGG	
NEUROD1	NM_002500.2	146	GCCCCAGGGTTATGAGACTAT<br>GAGAACTGAGACACTCGTCTGT	[2]
MAFB	NM_005461.3	146	TATAAACGCGTCCAGCAGAAGC<br>CCGGAGTTGGCGAGTTTCTC	
MAFA	NM_201589	195	CTTCAGCAAGGAGGAGGTCA<br>TTGTACAGGTCCCGCTCTTT	[2]
PREP1 (PKNOX1)	NM_004571.3	161	GGCTACACAGACATTAAGTATAGACAGC<br>GCTTGTCCACATCCATCGGG	
PBX	NM_002585.3	229	TTAAACTGCCACAGAATGAAGCCT<br>AGTTGTCTGAACCTGCCCCT	
PCSK1	NM_000439	117	AAGCAAACCCAAATCTCACCTGGC<br>TCACCATCAAGCCTGCTCCATTCT	
PCSK2	NM_002594	162	AAGATGGCTTTGCAGCAGGAAGGA<br>AGCCACATTCAAATCAAGGCCAGG	
Insulin	NM_000207.2	245	AGCCTTTGTGAACCAACACC<br>GCTGGTAGAGGGAGCAGATG	[4]
Glucagon	NM_002054.4	275	CATTCACAGGGCACATTCAC<br>CGGCCAAGTTCTTCAACAAT	[4]
Somatostatin	NM_001048.3	126	AGCTGCTGTCTGAACCCAAC<br>CCATAGCCGGGTTTGAGTTA	[4]
Pancreatic Polypeptide	NM_002722.3	180	ACCTGCGTGGCTCTGTTACT<br>CAGCGTGTCCTCTTTGTGTC	[4]

Ghrelin	NM_016362.3	156	AACACCAGAGAGTCCAGCA<br>CAACATCAAAGGGGGCGTT	
HPRT	NM_000194.2	148	TGTTGTAGGATATGCCCTTGACTAT<br>GCGATGTCAATAGGACTCCAGA	[2]

All primers were optimized for efficient (80-105%) amplification in a 2-step fast PCR reaction with a Tm of 62°C to generate a single peak during a melt curve and the expected product size.

References

1. D'Amour KA, Agulnick AD, Eliazer S et al. Efficient differentiation of human embryonic stem cells to definitive endoderm. **Nat Biotechnol**. 2005;23:1534-1541.
2. Gage BK, Webber TD, Kieffer TJ. Initial Cell Seeding Density Influences Pancreatic Endocrine Development During in vitro Differentiation of Human Embryonic Stem Cells. **PLoS ONE**. 2013;8:e82076.
3. Gage BK, Riedel MJ, Karanu F et al. Cellular reprogramming of human amniotic fluid cells to express insulin. **Differentiation**. 2010;80:130-139.
4. Li WC, Horb ME, Tosh D et al. In vitro transdifferentiation of hepatoma cells into functional pancreatic cells. **Mech Dev**. 2005;122:835-847.
