# Supplementary material for: The Role of ARX in Human Pancreatic Endocrine Specification
Source: PLoS One. 2015 Dec 3;10(12):e0144100. doi: 10.1371/journal.pone.0144100 (PMC4669132; doi:10.1371/journal.pone.0144100)
Supplement: S2 Table — Antibody concentrations, sources, and relevant staining conditions as applicable for this study. (PDF) [file pone.0144100.s003.pdf]

S2 Table. Antibody Sources and Conditions for Immunocytochemistry

Gene Name	Host Species	Supplier / Catalogue number	Staining Method	Dilution	Antigen Retrieval
CXCR4	Mouse IgG2A-PE	R&D Systems FAB170P	Fixed cell Flow	1:50	None
PDX1	Mouse IgG1k-PE	BD Biosciences 562161	Fixed cell Flow	1:50	None
NKX6.1	Mouse	DHSB F55A12	Fixed cell Flow	1:50	None
OCT4	Goat	R&D Systems AF1759	4% PFA fixed monolayer	1:500	None
SSEA3	Rat IgM	R&D Systems MAB1434	4% PFA fixed monolayer	1:250	None
Insulin	Guinea Pig	Sigma I8510	4% PFA fixed monolayer	1:500	None
Glucagon	Rabbit	Cell Signalling 8233P	4% PFA fixed monolayer	1:250	None
Somatostatin	Mouse	BCBC AB1985	4% PFA fixed monolayer	1:500	None
Chromogranin A	Sheep	Biomol International CA1128	Slide	1:200	HIER
ARX	Rabbit	Dr. P. Collombat Gift	Slide	1:500	HIER
PDX1	Guinea Pig	Abcam Ab47308	Slide	1:1000	HIER
PDX1	Rabbit	Dr. J. Habener Gift	Slide	1:1000	HIER
NKX6.1	Rabbit	Dr. A. Rezania Gift	Slide	1:500	HIER
PCNA	Mouse	BD Biosciences 610665	Slide	1:100	HIER
Insulin	Guinea Pig	Sigma I8510	Slide	1:1000	HIER
Glucagon	Rabbit	Cell Signalling 8233P	Slide	1:500	HIER
Somatostatin	Mouse	BCBC AB1985	Slide	1:1000	HIER
Somatostatin	Rabbit	Sigma HPA019472	Slide	1:1000	HIER
Ghrelin	Rabbit	BioVision 5991-100	Slide	1:200	HIER
Pancreatic Polypeptide	Goat	R&D Systems AF6297	Slide	1:200	HIER
NKX2.2	Mouse	DHSB 74.5A5	Slide	1:100	HIER
PAX6	Rabbit	Covance PRB-278P	Slide	1:250	HIER
ISL1	Goat	R&D Systems AF1837	Slide	1:25	HIER
PREP1 (PKNOX1)	Rabbit	Santa Cruz SC-6245	Slide	1:500	HIER
PBX1a/1b	Rabbit	Cell Signaling 4342	Slide	1:500	HIER

PC1/3	Rabbit	Lakshmi Devi<br>Gift	Slide	1:500	HIER
PC2	Rabbit	Affinity BioReagents<br>PA1-058	Slide	1:500	HIER
C-peptide	Guinea Pig	Abcam<br>Ab30477	Slide	1:100	HIER
eGFP	Mouse	Clontech<br>632375	Slide	1:500	HIER

HIER (heat induced epitope retrieval): 15 minutes at 95°C in 10 mM Citrate buffer with 0.05% Tween-20 pH 6.0.
SLIDE: PFA fixed, paraffin section of agarose embedded cell pellet or pancreatic tissue
BCBC (Beta Cell Biology Consortium)
DHSB (Developmental Studies Hybridoma Bank), University of Iowa
